# Advances and limitations for the treatment of spinal muscular atrophy

**DOI:** 10.1186/s12887-022-03671-x

**Published:** 2022-11-03

**Authors:** John W. Day, Kelly Howell, Amy Place, Kimberly Long, Jose Rossello, Nathalie Kertesz, George Nomikos

**Affiliations:** 1 Department of Neurology, Stanford University, Stanford, CA USA; 2 Spinal Muscular Atrophy Foundation, New York, NY USA; 3 Pfizer, Inc, New York, NY USA; 4 Casma Therapeutics, Inc, Cambridge, MA 02142 USA; 5 Scholar Rock, Inc, 301 Binney St, Cambridge, MA USA

**Keywords:** Spinal muscular atrophy, Survival motor neuron-1 gene, Survival motor neuron, Nusinersen, Onasemnogene abeparvovec-xioi, Risdiplam, Myostatin, Apitegromab, SRK-015

## Abstract

Spinal muscular atrophy (5q-SMA; SMA), a genetic neuromuscular condition affecting spinal motor neurons, is caused by defects in both copies of the *SMN1* gene that produces survival motor neuron (SMN) protein. The highly homologous *SMN2* gene primarily expresses a rapidly degraded isoform of SMN protein that causes anterior horn cell degeneration, progressive motor neuron loss, skeletal muscle atrophy and weakness. Severe cases result in limited mobility and ventilatory insufficiency. Untreated SMA is the leading genetic cause of death in young children. Recently, three therapeutics that increase SMN protein levels in patients with SMA have provided incremental improvements in motor function and developmental milestones and prevented the worsening of SMA symptoms. While the therapeutic approaches with Spinraza^®^, Zolgensma^®^, and Evrysdi^®^ have a clinically significant impact, they are not curative. For many patients, there remains a significant disease burden. A potential combination therapy under development for SMA targets myostatin, a negative regulator of muscle mass and strength. Myostatin inhibition in animal models increases muscle mass and function. Apitegromab is an investigational, fully human, monoclonal antibody that specifically binds to proforms of myostatin, promyostatin and latent myostatin, thereby inhibiting myostatin activation. A recently completed phase 2 trial demonstrated the potential clinical benefit of apitegromab by improving or stabilizing motor function in patients with Type 2 and Type 3 SMA and providing positive proof-of-concept for myostatin inhibition as a target for managing SMA. The primary goal of this manuscript is to orient physicians to the evolving landscape of SMA treatment.

## Background

Spinal muscular atrophy (SMA) is a rare, genetic neuromuscular condition causing progressive muscle wasting (atrophy) and weakness leading to loss of movement. Untreated SMA is often cited as the leading genetic cause of death in young children [1, 2]. The exact prevalence of SMA in the United States is not known with certainty and varies by type [3] (Table 1). An overall prevalence of SMA between one and two per 100,000 people has been suggested [4] with a frequency of 1/11,000 births [5]. Prevalence of SMA in the U.S. and European Union is estimated to be 30,000–35,000 cases [6], with an overall incidence estimated to be approximately 1/6000 to 1/10,000 births [4, 7–9].

**Table 1:** Spinal muscular atrophy prevalence [3]

Type	Birth Prevalence	Overall Prevalence
1	8.5/100,000	8,526
2	9.4/100,000	9,429
3	10.3/100,000	10,333

**Fig. 1:**
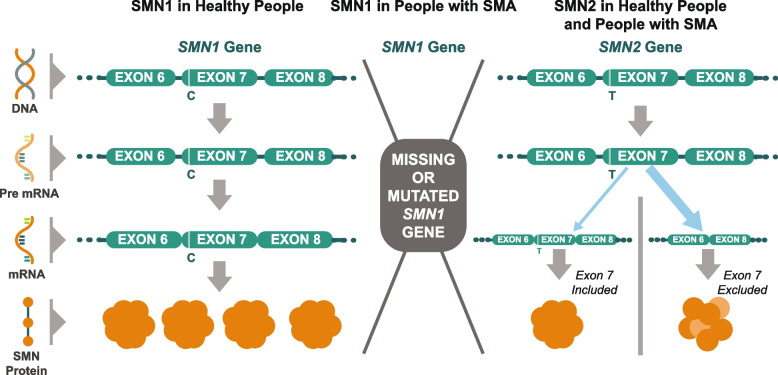
Normal SMN protein expression and in patients with spinal muscular atrophy. Survival motor neuron gene 1 (*SMN1*) encodes full length SMN protein needed to ensure survival of motor neurons and normal muscle growth and function (left). The nearly identical *SMN2* gene differs by only two nucleotides, a CT base change inside exon 7 that affects gene splicing and leads to exon 7 skipping in the majority of *SMN2* mRNA (messenger ribonucleic acids) (right). *SMN2* mRNA transcripts with exon 7 included provide a supplementary source of normal SMN protein; *SMN2* mRNA lacking exon 7 encodes truncated, rapidly degraded SMN protein. In patients with SMA, there is a homozygous deletion or loss of function of the *SMN1* gene, eliminating the body’s main source of SMN protein (center). The functional protein made by the *SMN2* gene is identical to that produced by the *SMN1* gene but is produced in insufficient quantity to support normal motor neuron functioning, muscle growth, and development. SMN1 codes for full length functional SMN1 protein which is the primary source of the SMN protein. SMN2 with exon 7 included is a full length, functional SMN protein (10–20%). SMN2 with Exon 7 excluded is an unstable rapidly degraded SMN protein (80–90%). Patients with SMN may have up to eight copies of the *SMN2* gene, all of which can produce limited quantities of SMN protein. Patients with more *SMN2* gene copies generally have less severe SMA [12–15]. Figure property of Scholar Rock, Inc

A homozygous deletion and/or mutation in the survival motor neuron-1 (*SMN1*) gene, localized on chromosome 5q, is responsible for the autosomal recessive disorder in more than 95% of cases [10]. 5q-SMA (hereafter referred to simply as “SMA”) phenotypes vary widely in severity, but all are associated with some degree of muscle weakness [8]. These mutations result in degeneration of motor neurons in the central nervous system (CNS) that may affect arm, hand, head and neck movement, crawling and walking abilities, breathing and swallowing [1]. Due to a preserved inverted duplication of a region on chromosome 5, there are two nearly identical *SMN* genes (*SMN1* and *SMN2*) [11]. *SMN1* expresses full length survival motor neuron (SMN) protein while the highly homologous *SMN2* gene expresses a small amount of full length SMN, but due to a splicing difference, it primarily expresses a shortened, unstable, and rapidly degraded isoform of the SMN protein [10] (Fig. 1).

The net effect of *SMN1* defects is diminished levels of full-length, stable SMN protein produced by *SMN2*. Complete absence of SMN is embryonically lethal, while diminished SMN content causes anterior horn cells to degenerate, ultimately resulting in motor neuron loss and subsequent skeletal muscle atrophy and weakness [16]. Although the *SMN2* gene can express small amounts of the full-length SMN transcript, the number of *SMN2* copies, which varies among effected individuals, affects disease severity, with more copies typically correlating with milder disease [17].

Proximal muscles are more highly denervated and atrophic compared to distal musculature in SMA [18]. Depending on the number of *SMN2* gene copies, symptoms can range from profound neonatal weakness with respiratory failure, often leading to death before the age of 2 years, to mild proximal lower extremity weakness in adulthood. These have been historically classified as Types 0 to 4 (Table 2); however, SMA classifications are changing due to newborn screening programs and the presymptomatic use of SMN restoration therapies. Patients are increasingly being diagnosed by newborn genetic testing, allowing for earlier restorative SMA treatment in presymptomatic infants.

**Table 2:** Historical spinal muscular atrophy subtypes [10, 19]

Type	Onset	Symptoms	Milestones
0	Prenatal	Respiratory failure at birth	
1	0–6 months	Severe deficits in motor function. Difficulties in breathing, coughing, and swallowing, fasciculations of the tongue	No sitting
2	< 18 months	Severe deficits in motor function. Delay in motor development, weakness, difficulties in coughing, joint contractures, scoliosis	Sitting, no walking
3	> 18 months	Variable degree of weakness, joint contractures, scoliosis, loss of ambulation	Independent walking
4	30 years	Variable, but milder weakness	Independent walking

Historically, untreated patients with Type 1 SMA had a 50% survival probability at 8–10 months of age and 8% survival at 20 months of age [3]. For patients with Type 2 SMA, the 1-year survival probability was 100%, decreasing to 82% at 10 years [3]. Overall survival of these patients is improving in the United States due to recently implemented newborn screening efforts [20], new therapies and presymptomatic treatment [21].

Since the introduction of new drug treatments for SMA, the observed disease trajectories differ significantly from the known natural history of the disease. The new phenotypes now cross the traditional subtypes of SMA (Table 2). For example, patients exhibiting symptoms at 6 months of age or younger (traditionally, SMA type 1) might achieve independent sitting (historically, SMA Type 2 by definition) if treatment is initiated early. It is now more appropriate to rely on a combination of age and functional status at start of drug treatment, age of symptom onset or number of SMN2 copies, rather than the traditional subtypes to define a clinical phenotype of SMA [19]. Despite these achievements, significant disability persists among patients treated after developing signs of SMA, including limited mobility, ventilatory insufficiency and difficulty swallowing [22].

With the availability of disease-modifying therapies, emerging therapeutic interventions, and on-going clinical trials of investigational compounds, it is also important to understand the natural history of the disease and identify new disease trajectories to better interpret patient response to treatment. Opportunities to maintain motor function throughout a patient’s lifetime as well as impact on fatigue measures, endurance, and patient-reported outcomes may also positively influence quality of life, shifting patient outcomes from survival to thriving [23].

### SMA newborn screening programs

Treatment is more successful if patients are treated presymptomatically, suggesting newborn screening is highly beneficial for this patient population [24]. It has been estimated that screening all newborns in the United States for SMA would find about 364 infants with the disorder annually, preventing up to approximately 100 children with SMA Type 1 from needing permanent ventilation and preventing up to approximately 68 deaths each year [25]. SMA was therefore added to the U.S. Federal Recommended Uniform Screening Panel (RUSP) for newborn screening in 2018 [26].

The RUSP is a list of disorders that the Secretary of the Department of Health and Human Services recommends for states to screen as part of their state universal newborn screening programs. Disorders on the RUSP are chosen based on evidence that support the potential net benefit of screening, the ability of states to screen for the disorder, and the availability of effective treatments. It is recommended that every newborn be screened for all disorders on the RUSP. Prior to 2013, the mean rate of prenatally diagnosed cases of SMA was 4.66 annually compared with 7.75 cases annually following population-wide screening [27]. As of June 2022, 46 states in the U.S. routinely screen newborns for SMA, testing 97% of all infants born in the country [28].

Screening is conducted using DNA extracted from dried blood spots with a multiplex real-time quantitative polymerase chain reaction assay targeting *SMN1* exon 7 which can be differentiated from *SMN2* exon 7 and is deleted in 95% of SMA patients [29]. SMA screening methods have high (100%) positive predictive value, and no false positives have been found when screening for deletions of exon 7 on both alleles [30]. Newborn screening is expected to increase the likelihood that pediatricians and family practice physicians will encounter patients with SMA. Additional information is available from the U.S. based organization, Cure SMA [31].

The European Alliance for Newborn Screening in Spinal Muscular Atrophy is striving for newborn screening programs in all European countries by 2025 [32]. Additional information is available from the organization, SMA Europe [33].

### Previous efforts to treat SMA

Numerous therapies that attempted to treat SMA, such as increasing the number of *SMN2* gene copies with hydroxyurea and increasing the level of full-length *SMN2* mRNA/protein with valproic acid, were carried forward through development in clinical studies. Alternative approaches for motor neuron survival such as olesoxime, a mitochondrial-targeted neuroprotective compound [34] or that improve contractility directly through calcium-sensitization in the sarcomere by activating skeletal muscle troponin with reldesemtiv (CK-2127107) [35] have also been attempted; however, due to not having been sufficiently effective or for other reasons, these aforementioned compounds are not being pursued further in clinical development for SMA at this time.

Advances in new treatments for SMA required an animal model of symptomatic SMA that replicated the human disease. A novel Δ7 mouse model of severe SMA demonstrates disease phenotype observed in human adolescent and adult SMA patients [36]. Using this model, preclinical studies showed treatment with an *SMN2* splicing modifier increased SMN protein and survival into adulthood with SMA-related disease pathology [36].

Numerous preclinical attempts at treating SMA were also unsuccessful. For example, follistatin is a myostatin inhibitor [37] but does not significantly enhance muscle development in neonatal SMA mice and did not ameliorate the SMA phenotype [38, 39].

### Approved therapies for treating SMA

As the expression of SMN protein is ubiquitous throughout the body, SMA can involve peripheral tissues in addition to motor neurons [40]. A multidisciplinary approach to treatment that includes, but is not limited to pulmonary, nutritional and orthopedic care [41], in combination with disease-modifying treatments. Three therapies that address the SMN-deficiency of SMA, referred to as SMN upregulators or SMN correctors, are FDA-approved and have received marketing approval in the European Union (E.U.).

#### SPINRAZA® (nusinersen) injection, for intrathecal use

**Fig. 2:**
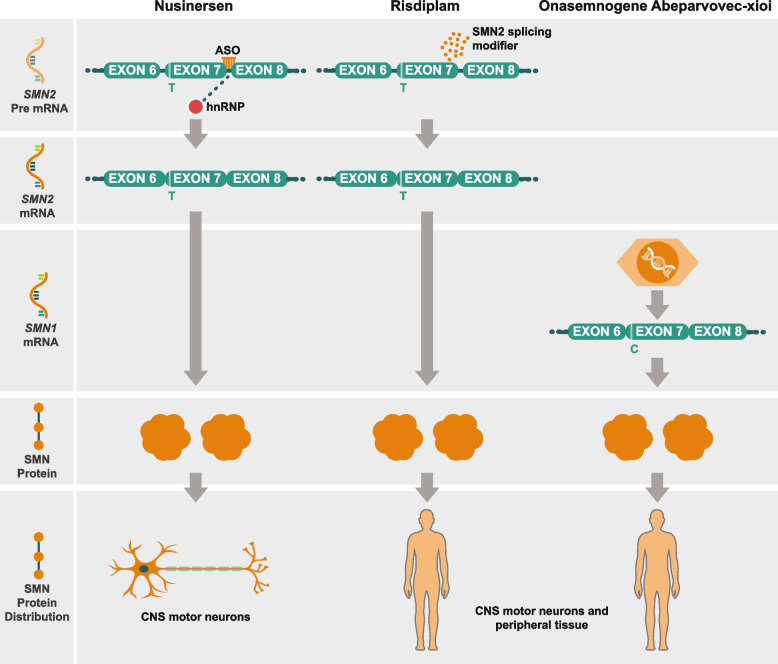
SMN correcting therapy, mechanism of action. The antisense oligonucleotide (ASO) nusinersen is an intrathecally-delivered splicing modifier that binds to the exon 7 silencer region on *SMN2* pre-mRNA (Pre mRNA) (left). By displacing the splicing repressor protein hnRNP, nusinersen promotes inclusion of exon 7 and boosts production of full-length *SMN2* mRNA. Functional SMN protein in central nervous system motor neurons is increased. Risdiplam is an orally available, selective small molecule that modifies SMN2 pre-mRNA (Pre mRNA) splicing (center). Risdiplam increases exon 7 inclusion in SMN2 mRNA transcripts and production of full-length SMN protein in the brain. This leads to increased production of functional SMN protein in the brain and throughout peripheral tissues. Onasemnogene abeparvovec-xioi is an adeno-associated virus 9 (AAV9)-based therapy that delivers a fully functional copy of *SMN* complementary deoxyribonucleic acid (cDNA) (right). Administered intravenously as a single-dose, the *SMN* transgene passes the blood–brain barrier and is introduced directly into target motor neuron cells throughout the CNS. Transduced cells produce full-length *SMN* mRNA transcripts, which enable continuous production of SMN protein in motor neurons and peripheral tissue over time [43–46]. Figure property of Scholar Rock, Inc

Nusinersen was approved in the U.S. in 2016 and the E.U. in 2017 for treating patients with SMA of all ages with 5q SMA based on the results of two phase 3 clinical trials. Nusinersen is an antisense oligonucleotide that modulates splicing of *SMN2* pre-messenger RNA to increase the proportion of full-length transcripts leading to higher levels of functional SMN protein [42] (Fig. 2).

Subjects in a randomized, double-blind, sham procedure-controlled study in symptomatic infants ≤ 7 months of age (*N* = 121) with genetically confirmed SMA and with symptom-onset before 6 months of age were randomized to receive intrathecal 12 mg nusinersen or a sham injection loading dose followed by active treatment or sham maintenance doses every 4 months [47]. Subjects were assessed by evaluating responders, i.e., subjects that achieved improvements in the Hammersmith Infant Neurologic Exam (HINE). The HINE evaluates seven different areas of motor milestone development, with a maximum score between 2–4 points for each developmental motor milestone. A total maximum HINE score is 26. A treatment responder was defined as a subject with a ≥ 2-point increase in ability to kick or a ≥ 1-point increase in the motor milestones of head control, rolling, sitting, crawling, standing, or walking [33].

Among the eligible subjects (*n* = 82), a significantly greater percentage in the nusinersen group (41%) were responders compared to the sham control group (0%). Among subjects in the final analysis (*n* = 81), the primary endpoint was time to death or permanent ventilation. Among nusinersen-treated subjects, there was a significant 47% reduction in the risk of death or permanent ventilation and a 63% reduction in the risk of death. Median time to death or permanent ventilation was 22.6 weeks in the sham-control group and was not reached in the nusinersen group [47]. The most common adverse events were lower respiratory infection and constipation, occurring in ≥ 20% of treated subjects but were attributable primarily to the underlying disease than to the treatment. The serious adverse event of atelectasis was more frequent among nusinersen-treated subjects than in control subjects (18% vs.10%) [47].

A second randomized, double-blind, sham-controlled study enrolled symptomatic subjects with later-onset SMA with symptom-onset after 6 months of age (*N* = 126) [47]. Subjects were randomized to receive an intrathecal loading dose of 12 mg nusinersen or sham injections followed by maintenance doses every 4 months. The primary endpoint after 15 months was the change from baseline Hammersmith Functional Motor Scale—Expanded (HFMSE) scores which evaluate motor function in subjects with limited ambulation, with a total possible HFMSE score of 66. It is comprised of 33 scored activities that give objective information on motor ability and clinical progression, such as the ability to sit unassisted, stand, or walk. Higher scores indicate better motor function.

Among nusinersen-treated subjects, the mean change in baseline total HFMSE scores was 3.9 versus -1 in the sham-treated group. The proportion of subjects who achieved a ≥ 3-point improvement in baseline total HFMSE scores was 56.8% in the nusinersen group versus 26.3% in the sham-control group. A 3-point increase in HFMSE scores represent improvements in two or three motor skills.

The most common adverse events occurring in ≥ 20% of treated patients and which occurred at least 5% more frequently than in control subjects were pyrexia, headache, vomiting, and back pain, consistent with the underlying SMA disease process and effects of lumbar puncture [47]. The intrathecal administration of Spinraza by lumbar puncture may require repeat sedation, depending on the clinical condition of the patient. Potential difficulties with this route of administration may occur in very young patients and those with scoliosis, which makes the use of ultrasound or other imaging techniques sometimes required; however, recent institutional interdisciplinary use of algorithms for selective use of image guidance can ensure safe and technically successful intrathecal administration [48–51].

#### ZOLGENSMA® (onasemnogene abeparvovec-xioi) suspension for intravenous infusion

Onasemnogene abeparvovec-xioi is an intravenously administered adeno-associated viral vector-based gene replacement therapy approved in the U.S. in 2019 for the treatment of pediatric patients who are < 2 years old with bi-allelic mutations in the *SMN1* gene [52]. It was approved for use in the E.U. in 2020. Onasemnogene abeparvovec-xioi gene therapy delivers a copy of the gene encoding human SMN protein in patients with SMA [53] (Fig. 2).

An open-label, single-arm, ascending-dose clinical trial assessed the safety and efficacy of onasemnogene abeparvovec-xioi in subjects < 2 years old with genetically confirmed bi-allelic *SMN1* gene deletions, two copies of the *SMN2* gene, and absence of the c.859G > C modification in exon 7 of the *SMN2* gene, and with SMA symptom-onset before 6 months of age. Onasemnogene abeparvovec-xioi was administered as a single intravenous infusion to low-dose (*n* = 3) and high-dose groups (*n* = 12).

After 24 months, one subject in the low-dose cohort required permanent ventilation while all subjects in the high-dose group were alive and without permanent ventilation. None of the subjects in the low-dose group were able to sit without support, stand or walk. In the high-dose group, nine subjects (75.0%) could sit without support for ≥ 30 s, and two (16.7%) could stand and walk without assistance. The most frequent adverse events with an incidence > 5 observed in four open-label studies of 44 subjects receiving intravenous (IV) infusion, were elevated aminotransferases exceeding the upper limit of normal (27.3%) and vomiting (6.8%) [52].

A phase 3 open-label, single-arm, single-dose trial enrolled symptomatic subjects < 6-months-old (*N* = 22) with SMA due to biallelic *SMN1* mutations (deletion or point mutations) and one or two copies of *SMN2* [34]. Subjects received a single 30–60 min IV infusion of onasemnogene abeparvovec-xioi (1.1 × 10^14^ vg/kg) and were then assessed once weekly for 4 weeks, and then monthly until age 18 months or early termination. Coprimary efficacy outcomes were independent sitting for ≥ 30 s (Bayley-III item 26) at 18 months of age and freedom from permanent ventilation at age 14 months. By the data cutoff, 13 of the 19 subjects continuing in the trial reached 14 months of age without permanent ventilation, one of the study’s coprimary efficacy endpoints.

In addition to survival, assessment of the other coprimary efficacy endpoints found that 10 of the 21 subjects (47.6%) achieved the ability to sit without support for ≥ 30 s between 9.2 and 16.9 months of age (mean age was 12.1 months). Based on the natural history of the disease, subjects who met the study entry criteria would not be expected to attain the ability to sit without support, and only approximately 25% of these subjects would be expected to survive (i.e., being alive without permanent ventilation) beyond 14 months of age. In addition, 16 of the 19 subjects had not required daily non-invasive ventilation (NIV) use.

Serious adverse events (n = 10, 45%) were most commonly consequences of the underlying disease, including some form of respiratory tract infection. Other events included transient transaminase elevation (n = 7, 32%), of which two (9%) developed severe elevation of transaminases that responded to steroids. Two subjects (9%) developed low platelet counts (≤ 75,000 /µL) that were not associated with clinical sequelae and resolved spontaneously [34]. The manufacturer has reported that 1,400 doses of *onasemnogene abeparvovec-xioi* have been administered worldwide since it received marketing authorization [54].

#### EVRYSDI® (risdiplam) for oral solution

Risdiplam (RG7916/RO7034067) is an orally administered, centrally and peripherally distributed small molecule that modulates *SMN2* pre-mRNA splicing to increase SMN protein levels (Fig. 2) [43]. It was approved for use in the U.S. in 2020 [43] and subsequently in the E.U. [55].

An open-label study assessed the efficacy, safety, pharmacokinetics, and pharmacodynamics of risdiplam in subjects with Type 1 SMA and symptom-onset between 28 days and 3 months of age (*N* = 21) [43]. Subjects were randomized to a high-dose group (*n* = 17) and had their dose adjusted to 0.2 mg/kg/day before 12 months of treatment while the low-dose group (*n* = 4) did not. Efficacy endpoints were the ability to sit without support for ≥ 5 s (Item 22 of the Bayley Scales of Infant and Toddler Development, 3^rd^ Edition [BSID-III] gross motor scale) and survival without permanent ventilation. Among subjects in the high-dose group, seven (41%) could sit independently for ≥ 5 s after 12 months of treatment and 19 (90%) were alive without permanent ventilation and reached ≥ 15 months of age. After ≥ 23 months of treatment, 17 subjects (81%) were alive without permanent ventilation and reached an age of ≥ 28 months. The most frequent adverse events reported in > 10% of these subjects were upper respiratory tract infections including nasopharyngitis, rhinitis, respiratory tract infections, pneumonia, constipation and vomiting [36].

The primary endpoint of a second randomized, double-blind, placebo-controlled study for Type 2 and 3 subjects aged 2–25 was the change in baseline Motor Function Measure 32 (MFM32) score after 12 months [43]. A key secondary endpoint was the proportion of subjects with a ≥ 3-point change in baseline MFM32 total score (maximum score 100) where a > 3 point change from baseline is considered clinically significant [56]. The MFM32 measures fine and gross motor function abilities that relate to daily functions from standing and walking to the use of hands and fingers.

Another key secondary endpoint was the Revised Upper Limb Module (RULM), a tool used to assess upper limb motor performance of SMA subjects that can capture progressive muscle weakness across the spectrum of the disease. Thresholds of improvement identified in previous studies as clinically meaningful are ≥ 2-point changes on the RULM (maximum score 37) [57].

The change in mean baseline total MFM32 score after 12 months was 1.36 in the risdiplam group versus -0.19 in the placebo group and the proportion of subjects with a mean change from baseline MFM32 total score ≥ 3 was 38.3% in the risdiplam group versus 23.7% in the placebo group. The change in mean baseline RULM total score was 1.61 in the risdiplam group versus 0.02 in the placebo group [57].

The most common adverse events reported in ≥ 10% of subjects treated with risdiplam and with an incidence greater than placebo-treated subjects were fever, diarrhea, and rash. Additional adverse events reported in > 5% of subjects and with an incidence > 5% more than placebo subjects were mouth and aphthous ulcers, arthralgia and urinary tract infection [43].

Although the effects of Evrysdi on fertility have not been investigated in humans, there is a potential effect on male fertility and women are advised to use contraception during Evrysdi treatment [43]. The effects of Evrysdi on the retinal structure observed in non-clinical studies has not been observed in clinical studies with SMA subjects [58]; however, long-term data are still limited [55].

Together, these new treatments (SMN-dependent therapies) address the genetic cause of the disease and have shown remarkable advances in SMA. In spite of these significant achievements, there remain unmet medical needs for this patient population.

### Limitations and unmet needs

Despite the strides made with transformative SMN-dependent therapies, uncertainties regarding treatment response and long-term outcomes for patients with SMA remain. The currently approved treatments offer a clinically meaningful therapeutic advance in patients with SMA; however, unmet needs remain for several reasons, some of which are described below.

#### Earlier treatment often leads to better outcomes

Recent research has demonstrated that abnormalities of motor axon development begin prenatally in infantile onset SMA patients and that these defects are associated with rapid postnatal degeneration of motor neurons [59]. These results suggest that minimizing treatment delay is essential to maximize therapeutic efficacy in patients. Indeed, it has been shown through numerous clinical trials and real-world evidence, that early treatment of SMA leads to better outcomes for patients [60]. For example, in the NURTURE trial, subjects treated presymptomatically with nusinersen, showed greater improvements in motor milestone scores in comparison to the treatment of symptomatic subjects with infantile-onset SMA in the ENDEAR study [48].

Older children and adults living with SMA, which represents two-thirds of the overall SMA population [48], may not have been treated early in their disease course due to lack of availability of treatments, clinical parameter restrictions, and/or age restrictions in drug labels. Additionally, the intrathecal route of administration required for nusinersen is particularly challenging for patients with contractures, scoliosis and spinal fusion, whereas risdiplam and onasemnogene abeparvovec-xioi may be currently limited to a certain age population [61]. Furthermore, patients with later-onset SMA who showed more modest improvements or stabilization in motor function in the CHERISH nusinersen clinical trial, may not have had the opportunity to demonstrate improvements and/or a stabilization of disease as those subjects who were treated earlier. Even for the patients treated early, questions remain whether sufficient SMN protein levels are achieved uniformly in all motor neurons to halt neurodegeneration, and whether the motor neuron dysfunction is fully reversible. For example, interim results from the ongoing NURTURE trial of nusinersen in presymptomatic subjects with SMA showed that even with early intervention, not all infants achieved age-appropriate milestones such as walking independently.

In addition, due to the degree of motor neuron loss and dysfunction at the time therapy is initiated, treated patients are vulnerable to progressive functional loss accompanying body and skeletal growth [62, 63].

While there have been impressive gains in survival, especially in presymptomatic patients, questions remain. Despite newborn screening, with differences dependent on the number of *SMN2* copies, delays in treatment, the lack of long-term data to confirm durability of effect especially through periods of growth and maturation, the safety and efficacy of gene therapy or repeated SMN-dependent therapy administration, questions on quality of life and cost-effectiveness must be considered. Less than half of patients in trials maintained an ability to thrive over the course of the treatment and based on the above limitations, a majority of patients may have residual deficits that may be mitigated with additional treatment options [64–73]. This is being investigated among patients with advanced disease using SMN upregulator combinations. Therapeutics that are independent of SMN upregulation may help improve outcomes for SMN-dependent treated patients that have not achieved maximum benefit [62, 63].

#### SMN upregulation outside the CNS and SMN-independent mechanisms

Although SMA is typically thought of as a disease of motor neurons, recent work has shown that SMN may play an important role in organs and peripheral tissues, outside of the CNS, particularly in muscles. Because nusinersen does not sufficiently cross the blood–brain barrier, the drug must be delivered intrathecally, limiting its exposure outside the CNS [61]. Similarly, though delivered systemically, it is not clear how well onasemnogene-abeparvovec-xioi transduces different cell types; further, because the virus does not integrate into a cell’s genome, it can be lost from replicating cells. Studies that follow patients for longer periods of time will help determine if patient outcomes are improved by systemic means, as opposed to restricted CNS restoration of SMN.

The pathophysiology of SMA extends beyond motor neuron function to include primary and secondary effects on muscle, pulmonary function, and other organs. The recently approved treatments for SMA, used singly or potentially in combination, may now fully restore SMN in all tissues and cell types, but there will still be unmet needs for most SMA patients in the magnitude of motor function improvement and the need to further restore muscle function. SMN-independent strategies may address these additional features of the disease and further improve motor function and general health [74, 75]. Although SMN-dependent therapies, do improve motor function, patients with SMA are not reaching the top end of motor function scores, and would ideally benefit from a two-pronged approach that targets the whole motor unit: treatments that optimize SMN restoration by directly affecting the motor neuron, and treatments that augment motor function by SMN-independent approaches through direct effects on the muscle [18, 41].

For example, among children with Type 2 SMA in the CHERISH study, data showed a clinically meaningful improvement in HFMSE scores after nusinersen therapy as their mean HFMSE scores increased from the low 20 s at screening, to mid- or high 20 s after 1–2 years of treatment [76, 77]. The relatively modest increase in mean HFMSE score in children with later-onset SMA may be due to the more difficult items on the HFMSE (i.e., squatting, jumping, stair climbing) are simply harder to achieve regardless of SMA type. Although the level of motor function improvement was deemed clinically meaningful, the low final outcome score highlights the need for additional enhancements to maximize motor function.

Patients with SMA experience limitations in mobility and daily activities associated with the progressive deterioration in motor function alongside emotional challenges including depression, anxiety, fatigue, social isolation, and a lack of effective interventions to address these aspects of quality of life (QoL) [23]. Pursuing alternative methods of treatment with differing mechanisms of action from the current therapies, may address this unmet need.

SMA also has a substantial and multidimensional burden on affected adults. While advances in supportive care and the new transformative treatments are rapidly reshaping the therapeutic environment, understanding the natural history, care pathways, and patient-reported outcomes associated with SMA in adulthood are critical to advancing research and clinical care.

In studies including patient-reported outcomes to-date, subjective well-being has not improved [78]. It has not been possible to identify a single treatment associated with statistically higher QoL; however, parents showed a trend toward the belief that their children with SMA have a greater QoL with current treatments compared to supportive care [78].

SMA remains a debilitating genetic disorder for many patients, despite the use of SMN upregulators; however, there are still unmet needs that demonstrate the importance of exploring SMN-independent mechanisms, specifically muscle-directed treatments that target the muscle component of the motor unit, used in combination with currently available treatments. Combining SMN restoration with SMN-independent treatment may address the varying degrees of muscle weakness, fatigue and immobility affecting SMA patients after receiving SMN upregulating treatment.

### Myostatin as a potential therapeutic target

**Fig. 3:**
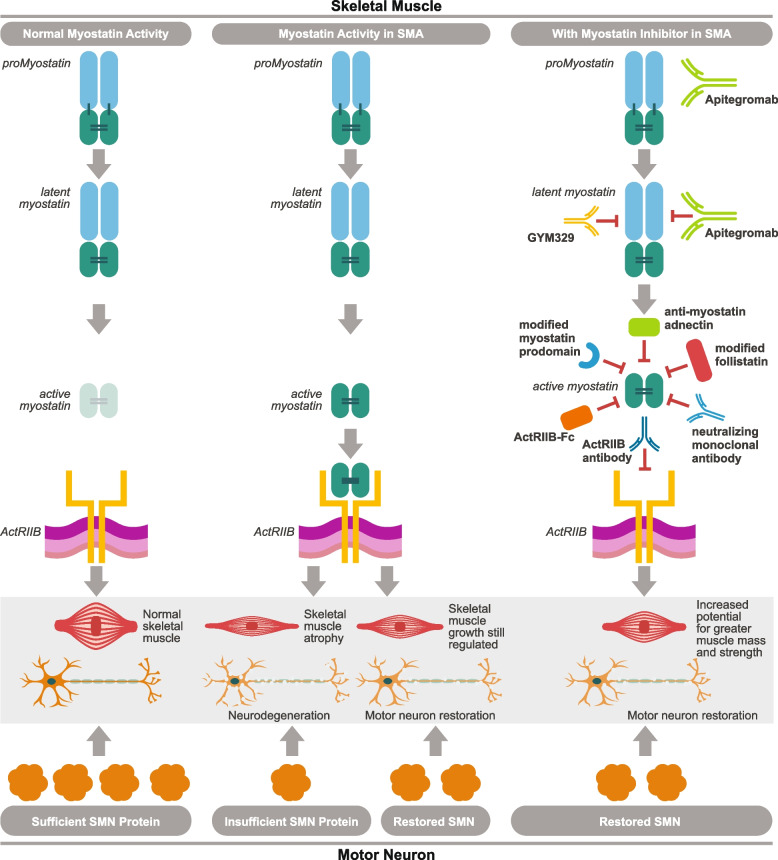
Myostatin inhibition MOA as add-on to SMN correctors in SMA. SMN protein promotes normal motor neuron function, which in turn provides the signals that activate and sustain muscle tissue (left). In SMA, insufficient SMN protein leads to degeneration of motor neurons and subsequent skeletal muscle atrophy (center). SMN correctors help to increase SMN protein production, stabilize neurodegeneration, and improve or maintain motor function, but may not return muscle to its normal size and function (right) Myostatin circulates as a complex of inhibitory prodomains. When the prodomains are proteolytically cleaved, the active myostatin dimer can bind to its receptor ActRIIB, The heterocomplex translocates to the nucleus where it regulates transcription. Several inhibitors of this signaling pathway have been developed including modified myostatin prodomain, modified follistatin, neutralizing monoclonal antibody and adnectin, ActRIIB-Fc, and ActRIIB blocking antibody. These strategies all lead to blocking myostatin binding to its receptor. Myostatin inhibition in combination with SMN correctors may directly address muscle atrophy and further restore motor function [12, 13, 82–84]. Apitegromab is a monoclonal antibody that selectively blocks the precursor, or inactive form of myostatin, blocking its activation in skeletal muscle. Myostatin is a negative regulator of skeletal muscle growth. Apitegromab specifically targets the upstream pro and latent forms of myostatin, which avoids cross-reactivity with other TGF-ß ligands and inhibits activation of myostatin. Apitegromab improves muscle mass and strength with fewer off-target effects and related toxicities than possible with less selective myostatin inhibitors [82, 85]. Figure property of Scholar Rock, Inc

Myostatin is a member of the transforming growth factor beta (TGF-β) superfamily of growth factors and is expressed primarily in skeletal muscle cells where it inhibits muscle growth [79] (Fig. 3). Since myostatin is a negative regulator of muscle mass, vertebrates lacking the myostatin gene are healthy but display increased muscle mass and strength [80]. In contrast, high levels of circulating myostatin are associated with muscle wasting in patients with cancer, HIV infection and other illnesses [81].

Numerous preclinical and clinical studies have demonstrated the potential role of myostatin in muscle atrophy [86], generating interest in myostatin as a promising therapeutic target for patients with muscle-wasting conditions, including SMA [87]. In multiple preclinical models of muscular atrophy, including SMA, myostatin inhibition is effective at maintaining muscle mass and function [88].

Pharmacokinetic studies showed maximum apitegromab serum concentrations were achieved 1-h postdose in adult rats and monkeys with relative dose-proportional accumulation of apitegromab at doses of 10 to 100 mg/kg. Apitegromab displayed a similar pharmacokinetic profile across animal species [89].

As apitegromab prevents the activation of mature myostatin in mice, pharmacodynamic studies showed dose-dependent accumulation of latent myostatin in the serum following repeated weekly IV administration of apitegromab at doses of 10 to 300 mg/kg in rats [89]. Administration of eight weekly doses of apitegromab to cynomolgus monkeys also resulted in a dose-dependent (but not dose-proportional) response in accumulated latent myostatin. These apitegromab-induced increases in serum latent myostatin which were observed in animals [90], healthy volunteers [91] and patients with SMA (see below), are considered indicative of target (latent myostatin) engagement with apitegromab and complex formation of latent myostatin with apitegromab in the muscle that is ultimately reaching systemic circulation and measured in the serum [92].

#### Phase 1 Clinical study

A phase 1 clinical trial in healthy, adult subjects was undertaken to assess the safety and tolerability of single and multiple IV doses of apitegromab. Secondary objectives were to assess the pharmacokinetics and immunogenicity of apitegromab, as well as to assess exploratory measures, such as the assessment of apitegromab pharmacodynamics [91].

During Part A, subjects received single, ascending doses of apitegromab ranging from 1 to 30 mg/kg as a 120-min intravenous (IV) infusion. During Part B, subjects were administered multiple, ascending doses of apitegromab 10, 20, or 30 mg/kg biweekly on Days 0, 14, and 28 as a 120-min IV infusion.

Serum latent myostatin displayed dose-dependent pharmacodynamics. Both single and multiple doses of apitegromab resulted in dose-dependent and sustained increases in serum latent myostatin, indicating robust target engagement.

The mean pharmacokinetic parameters after single IV infusions of apitegromab are summarized in Table 3 [91]. Serum apitegromab concentrations increased dose-proportionally and maximum plasma concentrations were observed within 8 h following the end of infusion. Apitegromab demonstrated linear, dose-proportional pharmacokinetics. Mean C_max_ values ranged from 25 μg/mL in the 1 mg/kg dose group to 744 μg/mL in the 30 mg/kg dose group. Apitegromab concentrations remained detectable for 112 days after infusion in all dose groups.

**Table 3:** Mean pharmacokinetic parameters after single IV infusions of apitegromab

Dose (mg/kg)	C_max_ (μg/mL)	T_max_ (hr)	AUC_(0-last)_ (hr*μg/mL)	AUC_(0-inf)_ (hr*μg/mL)	CL (mL/hr)	Vz (L)	t_1/2_ (hr)
1	25	6.0	11,647	12,748	6.05	6.8	786
3	83	4.7	33,097	35,037	7.69	6.9	624
10	278	3.4	105,973	126,053	7.29	5.4	543
20	555	4.7	216,171	227,308	7.10	5.7	588
30	744	5.3	347,298	367,866	6.60	5.9	623

*C*_*max*_ Maximum plasma concentration, *T*_*max*_ Time to peak plasma concentration, *AUC* Area under the curve, *CL* Clearance, *Vz* Volume of distribution, *t*_*1/2*_ Serum half-life

Adverse events observed for apitegromab were consistent with the underlying population and background therapy. The only adverse event occurring in more than one subject was headache (*n* = 3) and there were no clinically significant abnormalities or changes in vital signs, laboratory parameters, cardiac telemetry results, ECG results, or physical examinations. Immunogenicity, as evaluated by antidrug antibody testing, was negative for all subjects. The pharmacokinetic data support the potential for infrequent dosing. The results from this clinical trial and the preclinical studies supported further development and investigation of apitegromab in a phase 2 trial [91].

#### Phase 2 TOPAZ clinical trial

A recently completed phase 2 proof-of-concept clinical trial assessed the use of apitegromab for treating later-onset Type 2 and Type 3 SMA in pediatric and adult subjects, 2 to 21 years of age, with and without concomitant nusinersen therapy [93, 94]. The primary objectives were to evaluate safety and tolerability of apitegromab and efficacy by assessing changes in motor function outcome measures. Secondary objectives were to determine the time to therapeutic effect between low- (2 mg/kg) and high-dose (20 mg/kg) apitegromab and assess the immunogenicity of apitegromab. The overall study design is summarized in Table 4. Subjects received apitegromab every 4 weeks via IV infusion during the 52-week treatment period. Subjects were randomized into three groups:

• Nonambulatory subjects ≥2 years old treated with concomitant nusinersen initiated at or after age 5 years were randomized in a double-blind manner to receive apitegromab 2 mg/kg or 20 mg/kg
• Nonambulatory subjects 5 to 21 years old with concomitant nusinersen initiated after age 5 years received apitegromab 20 mg/kg.
• Ambulatory subjects 5 to 21 years old with or without concomitant nusinersen received apitegromab 20 mg/kg.

**Table 4:** TOPAZ trial – study design

	Ambulatory	Nonambulatory	Nonambulatory
Design	• *n* = 23; age 5–21 years	• *n* = 15; age 5–21 years	• *n* = 20; age ≥ 2 years
	• Open-label, single-arm	• Open-label, single-arm	• Double-blind, randomized (1:1) to 2 mg/kg or 20 mg/kg
	• 20 mg/kg SRK-015 IV Q4W	• 20 mg/kg SRK-015 IV Q4W	• SRK-015 IV Q4W
	• 12-month treatment period	• 12-month treatment period	• 12-month treatment period
Patients	• Ambulatory Type 3 SMA	• Type 2 or nonambulatory Type 3 SMA	• Type 2 SMA
	• Concomitant therapy with approved SMN upregulator (*n* = 12) or monotherapy (*n* = 11)	• Concomitant therapy with approved SMN upregulator started ≥ 5 years	• Initiated treatment with approved SMN upregulator before age 5 years
	• RHS Scores ≤ 63	• HFMSE Scores ≥ 10	• HFMSE Scores ≥ 10
Primary Objectives	• Safety	• Safety	• Safety
	• Mean change from baseline in RHS	• Mean change from baseline in HFMSE	• Mean change from baseline in HFMSE

*RHS* Revised Hammersmith Scale, *HFMSE* Hammersmith Functional Motor Scale Expanded

The nonambulatory subjects ≥ 2 years old randomized to high-dose apitegromab with nusinersen (*n* = 10) achieved improvements in baseline HFMSE scores by ≥ 3-points (*n* = 5, 63%), and ≥ 6 points (*n* = 5, 63%). Subjects receiving low-dose apitegromab (*n* = 10) achieved improvements in baseline HFMSE scores by ≥ 3-points (*n* = 5, 56%).

Nonambulatory subjects 5 to 21 years old (*n* = 14) achieved improvements in baseline (HFMSE) scores by ≥ 3-points (*n* = 4, 29%). Ambulatory subjects 5 to 21 years old (*n* = 23) achieved improvements in baseline Revised Hammersmith Scale scores by ≥ 3-points (*n* = 5, 22%) [69].

It should also be noted that any increase or stabilization of HFMSE score is a distinct and evident improvement over comparable natural history cohorts that have demonstrated progressive decreases in score over similar time frames [95, 96]. Among individuals with SMA Type 2 or 3 and their caregivers, slowing of disease progression and stabilization of disease course were considered clinically meaningful [95, 96]. A 3-point change in HFMSE scores is agreed upon by experts to represent a clinically meaningful change involving two or three skills [97]. A 6-point improvement reflects achievements in three to six skills. For example, improvements in motor skills include the following examples: trunk control when rolling and sitting and transitioning from lying to sitting or improved strength and consolidation of critical functions allowing for better maneuverability, transitioning and integration of proximal and distal functions that enable more advanced use of their well-developed fine motor skills such as sitting without support and hands and knees crawling [97, 98].

Apitegromab treatment in combination with nusinersen clearly showed benefits of apitegromab beyond the effects of nusinersen [91]. Exploratory analysis showed no correlation was observed between the duration of prior nusinersen therapy and 12- month HFMSE improvement.

Additionally, analyses of tertiary and exploratory endpoints showed both dosage groups, 2 mg/kg and 20 mg/kg, manifest early benefit, with a greater latency of the low-dose cohort further supporting apitegromab-attributable effects [94].

The doses explored in TOPAZ showed dose-dependent and dose-proportional increases in apitegromab exposure, with the high-dose achieving approximately tenfold increases in serum concentrations of apitegromab compared to the low-dose [74]. Both doses explored in TOPAZ, showed high target engagement, as measured by latent myostatin (> 100-fold increase from baseline) [93]. The higher 20 mg/kg dose offered relatively higher magnitude of target engagement. Scalable increases in HFMSE scores were seen following both high and low doses in combination with background chronic maintenance dosing with nusinersen. The 20 mg/kg dose increases in HFMSE were greater at all timepoints. Both the magnitude of target engagement and the magnitude of efficacy increased with increasing dose.

Incidence and severity of adverse events were consistent with the underlying patient population and background therapy. There was no evidence of immunogenicity. The most frequently reported treatment-emergent adverse events were headache, pyrexia, upper respiratory tract infection, cough, and nasopharyngitis [99].

TOPAZ demonstrated the potential clinical benefit of apitegromab by improving or stabilizing motor function in patients with Type 2 and Type 3 SMA and provided positive proof-of-concept for myostatin inhibition as an attractive target for managing SMA.

Since these positive TOPAZ results were obtained, other therapies that target myostatin signaling are now being explored in SMA, including the GYM329 antibody against latent myostatin, which in animal models increased muscle mass and improved grip strength in mice [44, 100]. Other ongoing SMA trials include a phase 3 apitegromab study in combination with nusinersen or risdiplam [NCT05156320​] [101], a phase 2/3 study of GYM329 in combination with risdiplam [102], a phase 2b/3 study of taldefgrobep alfa, an anti-myostatin adnectin [103], and a phase 1 of BIIB110, ActRIIA/B ligand trap [104, 105].

## Conclusion

Recent approaches to treating SMA have been highly effective in increasing SMN protein production by either modifying *SMN2* gene splicing with nusinersen and risdiplam, or *SMN* gene replacement therapy (onasemnogene abeparvovec-xioi). Despite these advances, unmet needs remain that include achieving age-appropriate milestones and treating the effects of SMA on peripheral tissues. Complementary approaches to tackling the whole motor unit, may provide even greater motor function benefits.

The recent introduction of newborn screening programs is identifying patients with SMA sooner, enabling early treatment referrals to SMA experts, who could recommend presymptomatic treatment; however, despite improvements in motor function with SMN-dependent treatments, there remain limitations of the current SMN-upregulating treatments that may contribute to unmet patient needs, that include achieving beneficial efficacy outcomes.

The monoclonal antibody apitegromab which blocks the activation of the negative regulator of muscle growth, myostatin, is in clinical development. Having achieved positive proof-of-concept, apitegromab has provided evidence of the potential to treat SMA and may represent a unique, SMN-independent approach, specifically targeting both forms of promyostatin; more specifically, a muscle-targeted therapeutic option for patients that still experience motor function deficits despite SMN protein-increasing therapy. Apitegromab, in combination with an SMN upregulator may further enhance motor function.

Apitegromab success has sparked an enormous interest in continued development of other myostatin inhibitors as potential therapeutic agents for treating SMA and are progressing to late-stage clinical trials in SMA. For example, novel agents such as the myostatin antibody GYM329 and taldefgrobep alfa are also in development for SMA in combination with SMN-correcting treatments and may provide additional therapeutic benefits for this patient population. A phase 3 apitegromab clinical trial for SMA is currently enrolling patients [SAPPHIRE; NCT05156320​].

Beyond treatments, the complexity of SMA also requires engagement of SMA patients with multidisciplinary teams to optimize outcomes through long-term follow up and monitoring of morbidities and to mitigate effects of potential safety concerns, ongoing proactive, supportive care to optimize mobility and to maintain maximum independence [39]. 

## Data Availability

Not applicable.

## Abbreviations

AAV9: Adeno-associated virus 9
AUC: Area under the curve
cDNA: Complementary deoxyribonucleic acid
CL: Clearance
C_max_: Maximum plasma concentration
CNS: Central nervous system
E.U.: European Union
HFMSE: Hammersmith Functional Motor Scale—Expanded
HINE: Hammersmith Infant Neurologic Exam
IV: Intravenous
MFM32: Motor Function Measure 32
NIV: Non-invasive ventilation
QoL: Quality of life
RULM: Revised Upper Limb Module
RUSP: Recommended Uniform Screening Panel
SMA: Spinal muscular atrophy
SMN: Survival motor neuron
*SMN1*: Survival motor neuron-1 gene
*SMN2*: Survival motor neuron-n gene
TGF-β: Transforming growth factor beta
t_1/2_: Serum half-life
T_max_: Time to peak plasma concentration
Vz: Volume of distribution
